# Using a cochlear implant processor as contralateral routing of signals device in unilateral cochlear implant recipients

**DOI:** 10.1007/s00405-021-06684-x

**Published:** 2021-02-22

**Authors:** Tom Gawliczek, Jérémie Guignard, Christoph Schmid, Wilhelm Wimmer, Marco Caversaccio, Martin Kompis, Stefan Weder

**Affiliations:** 1 Department of ENT, Head and Neck Surgery, Bern University Hospital, University of Bern, CH-3010 Bern, Switzerland; 2 Hearing Research Laboratory, ARTORG Center for Biomedical Engineering Research, University of Bern, Bern, Switzerland

**Keywords:** Contralateral routing of signals (CROS), Speech perception, Head shadow effect, Unilateral cochlear implant user

## Abstract

**Purpose:**

In unilateral cochlear implant (CI) recipients, a contralateral routing of signals (CROS) device enables to receive auditory information from the unaided side. This study investigates the feasibility as well as subjective and objective benefits of using a CI processor as a CROS device in unilateral CI recipients.

**Methods:**

This is a single-center, prospective cohort study. First, we tested the directionality of the CROS processor in an acoustic chamber. Second, we examined the difference of speech perception in quiet and in noise in ten unilateral CI recipients with and without the CROS processor. Third, subjective ratings with the CROS processor were evaluated according to the Client Oriented Scale of Improvement Questionnaire.

**Results:**

There was a time delay between the two devices of 3 ms. Connection of the CROS processor led to a summation effect of 3 dB as well as a more constant amplification along all azimuths. Speech perception in quiet showed an increased word recognition score at 50 dB (mean improvement 7%). In noise, the head shadow effect could be mitigated with significant gain in speech perception (mean improvement 8.4 dB). This advantage was reversed in unfavorable listening situations, where the CROS device considerably amplified the noise (mean:  – 4.8 dB). Subjectively, patients who did not normally wear a hearing aid on the non-CI side were satisfied with the CROS device.

**Conclusions:**

The connection and synchronization of a CI processor as a CROS device is technically feasible and the signal processing strategies of the device can be exploited. In contra-laterally unaided patients, a subjective benefit can be achieved when wearing the CROS processor.

## Introduction

In bilaterally deaf patients who do not sufficiently benefit from hearing aids, bilateral cochlear implants (CIs) are the treatment of choice nowadays. Hearing restoration of both ears has decisive advantages over unilateral implantation: the reduction of the acoustic head shadow, possibility of sound localization, availability of a backup device, bilateral summation effect, a better separation between the useful and interfering sound sources and the assurance that the better ear has been implanted [1–3]. Despite these advantages, there are medical and financial reasons why some unilaterally implanted patients with bilateral deafness might not be eligible for the implantation of the second side. These patients could benefit from a contralateral routing of signals (CROS) device. The CROS principle originates from conventional hearing aids and implies users wearing a microphone (or two microphones) on the non-implanted side. The signal is sent contra-laterally to the CI processor. While in the hearing aid community, CROS devices have been established for a long time, in CI patients, the connection of a CROS device with the main implant is not yet routinely used.

Previous studies show that CI recipients might benefit from a CROS system especially in favorable hearing situations when speech is presented on the CROS side [3–6]. In unfavorable situations (when noise is presented on the CROS side), the study results are not as consistent. Whereas most findings reported a slight deterioration in speech intelligibility [5, 7, 8], in a diffuse noise field, there was no significant difference [3]. Regarding subjective results, most studies demonstrate only small and non-significant effects with a switched-on CROS device. Mosnier et al. demonstrated in a long-term study that, despite study participants wearing the CROS device regularly over a one year period and gave positive feedback, subjective ratings of three different questionnaires could not statistically capture this effect [9].

One reason that CROS devices are not yet widely used in combination with implants is that only a minority of CI manufacturers offer commercially available CROS devices [9, 10]. If there is no compatibility, patients depend on self-developed solutions [4], which are neither certified nor will they be reimbursed. Implant manufactures have heavily invest to improve the transmitter technology and connectivity of the CROS devices over the last years [9]. However, the potential benefit of the same signal processing strategy as in the sound processor on the implanted side have not yet been investigated so far.

This study investigated the feasibility of connecting a CI processor as CROS device in unilateral CI users. We hypothesized that the scene classifier and automatized microphone directionality would result in objective and subjective benefits. Our approach included three steps. First, we performed a technical evaluation of the synchronicity, summation and directionality effect of the CROS processor. Second, we examined the difference of speech perception thresholds in implanted people when wearing the CROS processor. And third, we asked patients to evaluate the subjective benefit of the CROS device in their everyday life during a trial period of two weeks.

## Methods

This single-center, prospective study was approved by the local institutional review board (KEK-BE, No. 165/11). All study participants gave written informed consent before starting the study procedure.

### CI-CROS setup and technical evaluation

As CROS processor, we used a CP910 Cochlear Nucleus 6 System (Cochlear Ltd. Sydney, Australia). The processor was worn behind the ear in the usual manner, but without an antenna. The CI and CROS processors were connected by custom-made bilateral connection cables that were plugged into the accessory sockets. They were symmetrically designed and could be used in either direction. All cables were tested before use. The specially designed firmware allowed the CROS processor to continuously transmit the audio signal to the CI side. Signals from the CI and CROS sides were mixed at a ratio of 1:1. The audio processing strategies of the CROS processor were identical to those of the CI processor and were set to "SCAN" (*n* = 6) or "Standard" (*n* = 4) to match the setting of the CI processor for each patient. “SCAN” is the name of the scene classifier that activates microphone directionality algorithms when appropriate [11]. “Standard” is a fixed microphone directionality.

First, we measured the signal delay caused by the cable connection of the two processors. Second, to test the output of three different setups (CI alone, CROS processor alone, CI-CROS processor), we presented a narrow-band noise centered at 1 kHz calibration signal at 65 dB SPL coming from the front. The combination of the signals was recorded via a custom cable connected to the processor’s built-in acoustic output socket on the CI side. The acoustic output was programmed to deliver the input signal without amplification and with a flat response on the whole frequency spectrum. The output was recorded with an Audio Analyzer (Rhode & Schwarz, Gemany). Third, we evaluated the directionality effect of the CI and CROS processor in an acoustic chamber (6.0 × 2.2 × 4.1 m^3^, frequency-independent reverberation time of 0.14 s) with a head and torso simulator (Brüel & Kjaer, Germany).

### Study population and test procedure

Ten German-speaking unilateral CI users using the Cochlear Nucleus System (Cochlear Ltd. Sydney, Australia) were included (5 females, 5 males, aged 48–79 years, mean age 64 years). All participants were experienced CI recipients implanted at least two years before the start of the study. All of them had good speech understanding with the CI (≥ 75% understanding of monosyllabic words at 65 dB SPL).

Study participants had two appointments. On the first visit, the CI-CROS system was adjusted and participants were instructed in the handling of the device. We advised to wear the CROS system during the entire day for at least two weeks. At the control visit, all participants stated that they had followed these instructions. During the trial period at home, participants filled out the client oriented scale of improvement (COSI) [11]. The questionnaire includes 11 hearing situations (e.g., “following conversations with 1 or 2 in quiet” and “familiar speaker on the phone”) and 4 descriptions about the emotional state (e.g., “feeling left out” and “feeling upset or angry”). Listening situations and emotional descriptions must be ranked on a scale of −5 (much better with the CI only) to + 5 (much better with the CI-CROS system). At the beginning of the trial period, participants were given a booklet with the COSI hearing situations and emotional descriptions. All questions were studied before starting the experiment. We instructed participants to answer at least one question per day. As listening situations may vary depending on the day, study participants were able to choose the question to be answered. In addition, study participants were able to add notes under each question. At the follow-up visit, the questionnaire was re-examined with the investigator and any questions noted were clarified.

As half of the study participants used a conventional hearing aid in the non-CI ear outside the study (which was not used during the trial period), for the analysis of subjective ratings, two subgroups were formed accordingly: Individuals without a hearing aid on the non-CI ear (group non-HA, *n* = 5; range of pure tone average (PTA): 84 to 120 dB HL) and individuals who normally wore hearing aids (group HA; *n* = 5; range of PTA: 49 and 116 dB HL).

On the second visit, speech understanding was evaluated in an acoustic booth. For speech perception in quiet, we employed the German Freiburg monosyllabic word test at 50 and 65 dB SPL [12]. Speech perception in noise was assessed using an adaptive German matrix test (Oldenburger Satztest—OLSA) [13]. During the OLSA test, a quasi-diffuse noise (N_D_) at 65 dB SPL was presented by 4 loudspeakers (JBL, USA) arranged in 90-degree intervals with 1 m distance to the listener [3]. Sentence tests were presented from 3 different spatial configurations: speech from the front (S_0_N_D_), speech from the CI side (S_CI_N_D_), and speech from the CROS side (S_CROS_N_D_). For data visualization and analysis, we used GraphPad Prism (GraphPad Software, version 8.4.3 Inc., USA).

## Results

When analyzing the objective hearing results and surveys, we focused on descriptive statistics of the collected data due to the relatively small sample size of 10 participants.

### CI-CROS setup and technical evaluation

**Fig. 1:**
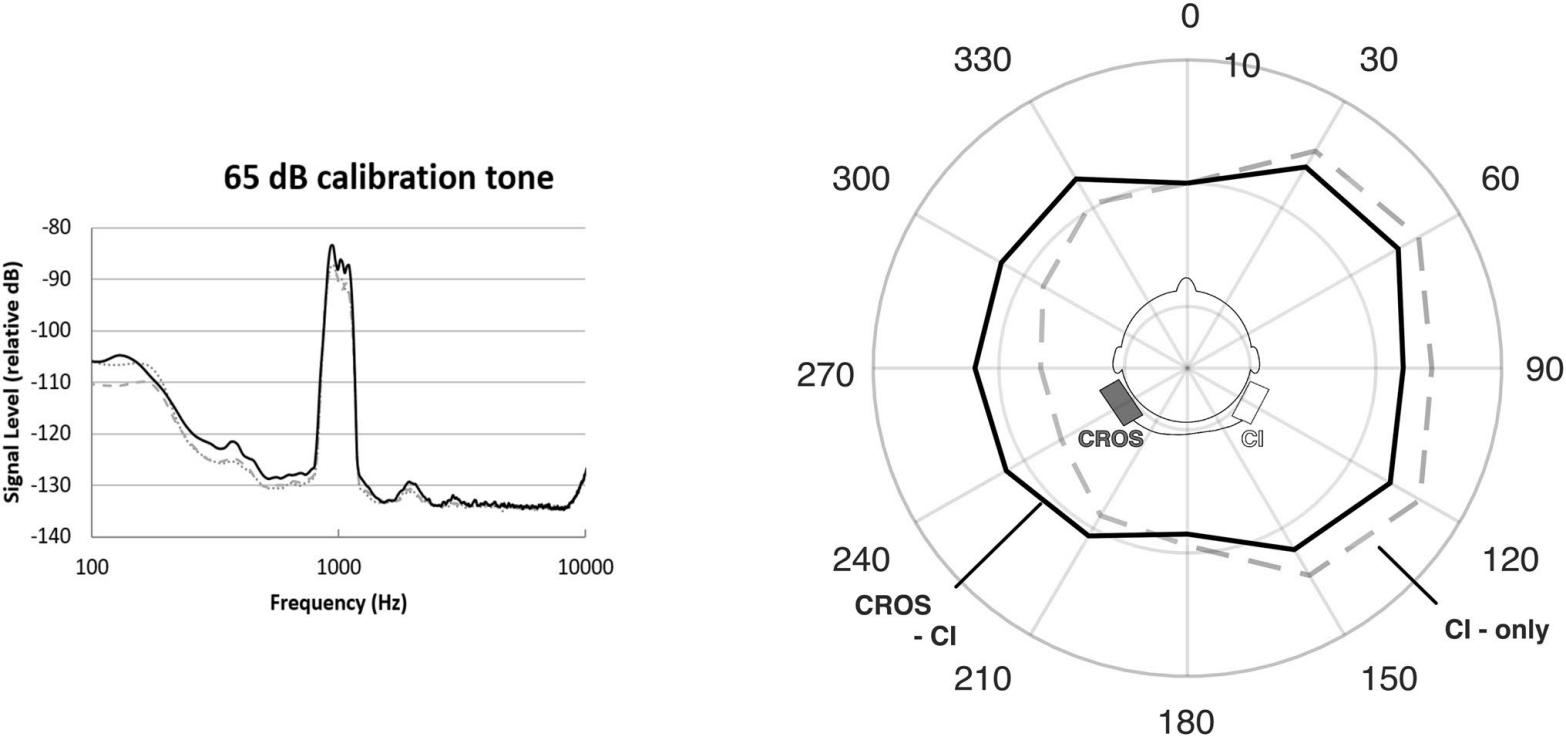
Using a narrow-band noise centered at 1 kHz calibration signal at 65 dB SPL, the left panel shows the output calibration of the satellite processor (dashed line), the CI processor (dotted line) and both connected devices (CROS, black line). The polar plot on the right shows the directionality effect in dB measured on a Kemar simulator in “Standard” mode in an anechoic chamber. The added CROS processor (solid line) led to a more uniform amplification along all azimuths compared to the CI processor only (dashed line). Both lines are the average of measurements at frequencies 0.25, 0.5,1,1,2,4, kHz

Using the cable connection, the bilateral link delay was 3 ms. The frequency spectrum in response to the calibration signal of the CI and CROS processor was very similar (Fig. 1). When the signals of the CI and CROS processor were mixed, there was a 3 dB signal increase for signals from the front. Using the Kemar simulator, the compensation of the head shadow by the additional CROS processor was observable.

### Speech understanding

**Fig. 2:**
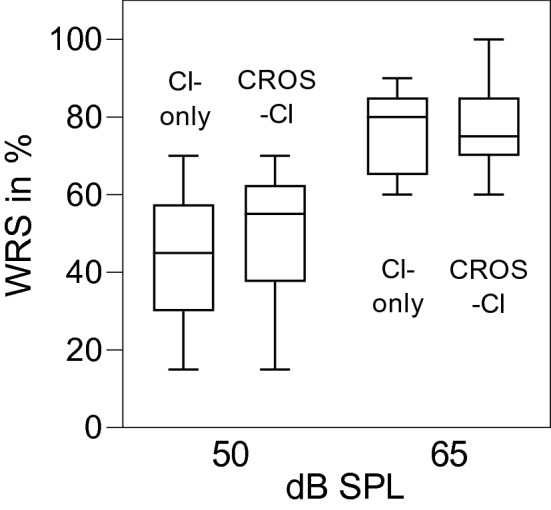
Monosyllabic word recognition scores at 50 and 65 dB SPL. Whiskers are minimum and maximum values, boxes are interquartile ranges and lines are the median

With the activated CROS device, speech perception thresholds in quiet slightly increased at 50 dB SPL (Fig. 2; mean 43% without additional CROS device, 50% with the CROS device). With a higher presentation level (65 dB SPL), this effect leveled out (mean 75% without additional CROS device, 78% with the CROS device).

**Fig. 3:**
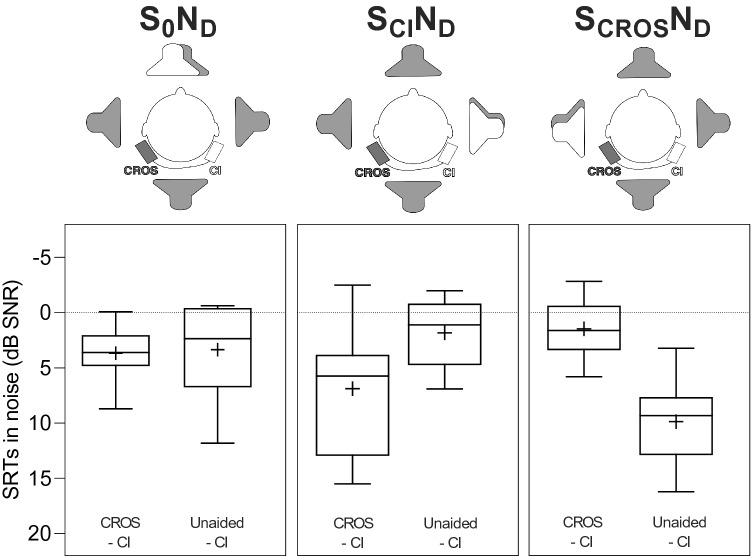
Speech reception thresholds (SRT) in a quasi-diffuse noise field (N_D_) with speech presented from the front (S_0_), from the CI (S_CI_) or from the CROS side (S_CROS_). Whiskers are minimum and maximum values; boxes are the interquartile ranges; the middle line corresponds to the median and the “ + ” to the mean

For speech perception in noise, with the additional CROS processor, the greatest benefit was obtained when the signal was presented to the non-CI side (Fig. 3; S_CROS_N_D;_ mean improvement 8.4 dB). However, when the signal was applied at the CI side, on average, speech perception decreased by 4.8 dB SNR. For speech perception from the front, patients showed an almost equal performance with or without the activated CROS processor (mean difference – 0.3 dB).

### Subjective benefit

**Fig. 4:**
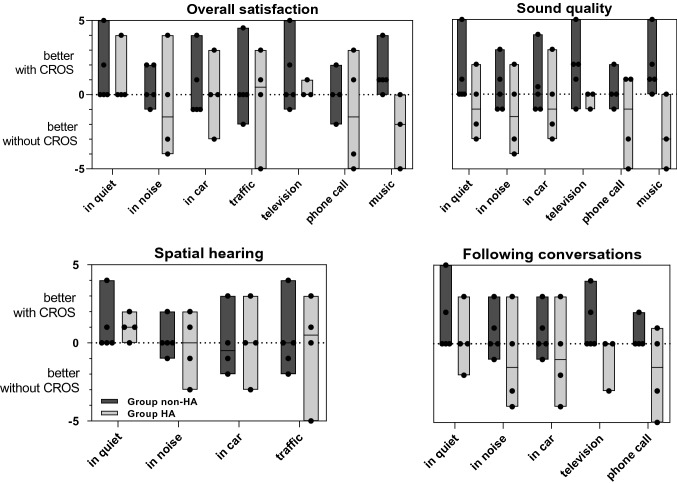
Subjective ratings according to the COSI questionnaire. Subjects were grouped according to the usual fitting of the non-CI ear. In contrast to group A (dark gray bars), individuals from group B (light gray bars) usually wore a contralateral hearing aid

Figure 4 displays the subjective ratings grouped in participants who usually do not wear hearing amplification on the non-CI side (group non-HA) and participants who do wear a hearing amplification on the non-CI side (group HA). Overall, most subjects in group non-HA were satisfied with the additional CROS device. The biggest improvements in sound quality were measured when watching television (median = 2) and listening to music (median = 1). In contrast, car journeys resulted in a deterioration in overall assessment (median =  – 1) and spatial hearing (median =  – 0.5). Contrary to our expectations, a deterioration was also observed when the CROS device was directed towards the passenger. This could be explained by a reverberation effect, which is created in a small driver's cab and, thus, leads to an amplified background noise.

In group HA, the satisfaction with the CROS device was considerably lower. Although some subjects (2 out of 5 participants) felt a benefit through the additional CROS device, others felt a deterioration in many hearing situations. Especially, a deterioration in ratings occurred during conversations in noise (median =  – 1.5), using the telephone (median =  – 1.5), and in confined spaces (e.g., in a car).

Regarding emotional states and social aspects, the idea of an additional CROS device was well tolerated by all the subjects. Overall, the patients who were already accustomed to a hearing aid on the non-CI side were more familiar with the CROS device than the non-hearing aid group.

## Discussion

Our prospective study explored the feasibility and outcome of connecting a CI processor as a CROS device in unilateral CI recipients. Our study setup had the following advantages: as CI and CROS processor used the same hardware and signal processing strategies, the devices could be matched and evaluated. By assessing the signal processing circuits, we could set the mixing ratio to exactly 1:1 and evaluate the performance, directionality, and signal delay with and between the two devices. Further, participants could continue to use their preferred directionality setting, which, during the trial, was adopted by the CROS device, making a direct comparison to their usual fitting possible.

In our findings, we could show that the connection and synchronization of a CI processor as a CROS device is technically feasible and the automatic scene classifier and microphone directionality of the device can be exploited. By its use, (i) the head shadow effect can be mitigated with a significant gain in speech perception compared to our previous study [3], (ii) a more constant amplification along all azimuths can be attained, and (iii), depending on patient characteristics, a subjective benefit can be achieved.

### Technical evaluation of the CROS processor

The connection of two devices without time delay remains a challenge. In our test setup, there was a delay of 3 ms. This short delay time could only be achieved using a cable connection. Recent publications have mostly employed a wireless connection of the CROS device [9, 10] resulting in significantly longer delay times. With the 3 ms delay, our figures still showed a comb filter effect for signals from the front. In real-life situations, we anticipate this effect to be much smaller as (i) the reverberation paths of natural sounds vary according to their environment. [14] and (ii) the comb filtering quickly disappears at different stimulation angles due to the head shadow effect. However, the technical advantage of a cable connection may not outweigh the practicality and aesthetics aspects of a wireless connection. Furthermore, the problem of longer delay times could be solved by signal processing strategies. The CI and CROS signal could be delayed in such way that the devices are synchronous.

In our figures, the measured output of the CI and CROS processor had the same spectral characteristics as the CI-only signal. When both, CI and CROS processor signals were mixed, there was a 3 dB signal increase. This is expected due to signal summation and could provide a performance benefit for signals from the front. Lastly, with the switch-on of the CROS processor, the polar plot showed a more constant sensitivity across all azimuths compared to the CI-only situation; the amplification on the CI side was shifted slightly to the opposite side with suppression of the head shadow effect.

### Speech understanding

At 50 dB SPL, speech perception in quiet was slightly better when the additional CROS processor was activated. At louder stimulation levels (65 dB SPL), this effect leveled out. This finding could be due to the above-mentioned summation effect which is more pronounced at lower presentation levels [15, 16].

In noisy situations and consistent with existing literature, the CROS processor amplified speech signals when they were applied to the non-CI side (median improvement 8.4 dB SNR) [9, 17]. It is interesting to note, that a gradual increase of the signal to noise ratio seems possible over time due to a learning effect [9]. However, our experiment was too short to allow such learning curve. In our figures, in the unfavorable situation S_CI_N_D_, we observed that the CROS processor amplified the noise. This stands in contrast to earlier findings with the same study setup where the quasi-diffuse noise was not amplified by the additional CROS device [3]. Lastly and as expected, when speech signals came from the front, there was only a small difference whether the additional CROS processor was added or not.

Study findings from different research groups are summarized in Table 1. It must be taken into account that study protocols varied and, thus, the results cannot be compared directly. In our current figures and in comparison to our earlier findings [3], there was a significant improvement in the S_CROS_N_D_ situation and a concurrent deterioration in the S_CI_N_D_ situation. This can be explained by the fact that the directionality settings of the currently used system amplify lateral signals. This leads to an improved speech understanding in the situation S_CROS_N_D_, but also to an increased deterioration in situation S_CI_N_D._ With a fixed directional microphone setting to the front, we would expect less pronounced differences.

**Table 1:** Overview of studies investigating speech understanding in noise with CI and CROS devices

First author	Year	CROS system	Number of subjects	Connection	Signal processing	Test setup	Value	Speech front	Speech CI	Speech CROS
Gawliczek	2020	Cochlear nucleus 6 (Cochlear)	10	Wire	Yes	Diffuse	Median Mean	– 1.25 – 0.3	– 4.6 – 4.8	7.7 8.4
Mosnier	2019	Naída link (Advanced bionics)	13	Wireless	Yes	S_0_N_CI,_ S_CROS_N_CI_	Median	2.7	N/a	8.0
Ernst	2019	Naída link (Advanced bionics)	10	Wireless	Yes	S_0_N_Diff (30:330)_, S_cros_N_CI_	Median	4	N/a	4.15
Snapp	2019	Naída link (Advanced bionics)	12	Wireless	Yes	S_0_N_0,_ S_CI_N_CROS_	Median	2.5	– 3	9.75
Wimmer	2017	iLapel Microphone (Phonak)	12	Wire to a wireless transmitter	No	Diffuse	Mean	1.9	< 0.5	1.7
Taal	2016	Ambra (Phonak)	5	Wireless	No	Diffuse	Mean	– 1.6	– 4.1	10.5
Weder	2015	Croslink receiver CRX (Phonak)	13	Wire	No	S_0_N_CROS_, S_CROS_N_0_	Median	– 0.7	N/a	6.4
Van Loon	2014	Lapel microphone (Cochlear)	15	Wire	No	S_0_N_0_, S_CROS_N_CI,_ S_CI_N_CROS_	Mean	– 1.4	– 5.7	6.7

The last three columns show the difference of speech reception thresholds for the test setups "speech from the front", "speech from the CI side", and "speech from the CROS side". Positive values indicate an improvement, negative values a deterioration with the additional CROS device

### Subjective benefit

We observed a difference between the non-HA and HA group. Patients who only had a CI without an additional hearing aid were generally satisfied with the idea of a CROS device. This was reflected in the subcategory’s overall satisfaction, sound quality and following conversations. Our study results stand in contrast to findings of [9] where two different questionnaires (i.e., the Abbreviated Profile of Hearing Aid Benefit [18] and the abbreviated Speech Spatial Qualities Questionnaire [19]), did not show a difference when using the CROS device. The explanation for these controversial findings could be diverse. Most apparent, we used a questionnaire that focuses on specific everyday listening situations. Participants had to fill in at least one hearing situation per day but could decide for themselves which one it was (depending on acoustic conditions during a day). Regarding the technical configurations, our system was technically evaluated before use. A certain time delay between the two devices was present but was shorter compared to wireless systems. Lastly, the sound processing strategy of the CI and CROS processor was identical, which significantly improved the interaction between the CROS device and the CI processor.

In our experiment, in noisy environments and in rooms with a lot of reverberation, there was an adverse perception with the additional CROS device in group non-HA and HA. These situations are most challenging for a CROS device [4, 8]. The problem could be solved by a so-called mute button, which is already successfully used in certain devices [9]. This feature seems to work well in noisy situations and can significantly improve the performance of CROS devices.

In the group HA, individuals did not experience satisfaction with the CROS processor in many listening situations. This was expected, since the combination of electric and residual acoustic hearing has proven to be beneficial in many studies [20–22]. In the future, in the subgroup of patients with residual hearing in the non-CI ear, an electro-acoustic CROS fitting could be technically feasible; the non-CI ear could be supplied with acoustic signals while at the same time the signal could be transmitted contra-laterally to the CI.

## Limitations

In our study, the connection between the sound processors was achieved by a wired connection to equalize both processed input signals with minimal delay time. However, for aesthetic reasons, a CROS device with a wireless connection using identical signal processing strategies could increase the acceptance of these systems.

The sample size and observation period of our study were limited. Larger samples are necessary, especially for subjective data. In subgroup HA, there was a bias regarding subjective ratings. As these subjects were used to wearing a conventional hearing aid on the non-CI side, the poor subjective outcome was rather due to the absence of the hearing aid on the non-CI side than to non-functionality of the CROS processor.

Finally, the SCAN setting uses dynamic microphone directionality. Therefore, dynamic test systems could possibly better capture the responses of the audio processor to complex listening situations [23, 24].

## Conclusion

The connection and synchronization of a CI processor as a CROS device is technically feasible and the signal processing strategies of the device can be exploited. Depending on patient characteristics, a subjective benefit can be achieved. Since a CROS device can be connected and tested very easily, it could be offered as an aid to bilaterally deaf patients who wear only one CI. In the future, advanced sound processing strategies could further increase the acceptance and benefit.
